# Utilizing a Sarmiento Brace to Attain Union of a Humeral Shaft Fracture Nonunion With Hardware Failure

**DOI:** 10.1155/cro/9111506

**Published:** 2026-04-25

**Authors:** Dylan J. Cannon, Brandon R. Hull

**Affiliations:** ^1^ College of Medicine, Department of Orthopedic Surgery and Rehabilitation, University of Oklahoma, Oklahoma City, Oklahoma, USA, ou.edu

**Keywords:** case report, fixation failure, humeral shaft fractures, humerus nonunion, Sarmiento brace

## Abstract

Humeral shaft fractures are prevalent, with the majority receiving nonoperative treatment using a functional brace. However, some patients necessitate surgical intervention either as an initial approach or in cases where nonunion arises despite conservative management. This case report presents a patient who initially sustained a closed humeral shaft fracture managed nonoperatively with a Sarmiento brace. Unfortunately, she progressed to nonunion, prompting the need for surgical intervention via open reduction and internal fixation. Following surgery, she experienced hardware failure but ultimately achieved union after returning to her Sarmiento brace.

## 1. Introduction

Humeral shaft fractures are common injuries and account for 1%–3% of all fractures [1–4]. They are observed in a bimodal distribution, predominantly in young males after high‐energy trauma and elderly females after low‐energy falls [1, 3, 4]. Nonoperative management is typically indicated for closed fractures with acceptable alignment, which has been established as less than 20° of anterior or posterior angulation, less than 30° of varus or valgus angulation, less than 15° of rotational malalignment, and less than 3 cm of shortening [5, 6].

The introduction of the functional brace, commonly referred to as the Sarmiento brace, has demonstrated that nonoperative management can be effectively utilized in most cases. Patients can expect fracture union and a good functional outcome with proper application of the Sarmiento brace [7–10]. Surgical intervention is typically required via open reduction internal fixation for patients that fail the Sarmiento brace [6, 10, 11]. Nonunion after surgical management of humeral shaft fractures is typically treated with revision surgery, often with bone grafting.

Risk factors for humeral shaft nonunion in nonoperatively treated fractures include poor bone quality, elevated body mass index (BMI), body habitus including a heavier upper body, NSAID use, advanced age, and greater initial fracture displacement [12, 13]. Driesman et al. found that mobility at the fracture site 6 weeks after injury predicted nonunion with 82% sensitivity and 99% specificity [14]. Nonunion cases are typically caused by inadequate stability and are treated with open reduction and internal fixation. Nonunion of surgically treated humeral shaft fractures can occur in up to 10% of cases and is often due to inadequate mechanical stability, poor vascularity at the fracture site, infection, and higher American Society of Anesthesiologists (ASA) score [15–19].

We present a case involving a patient who sustained a humeral shaft fracture that was treated nonoperatively in a Sarmiento brace. The patient failed to unite and underwent surgical stabilization. Failure of fixation occurred and the patient was indicated for revision fixation. The patient subsequently went on to heal her fracture by going back into the Sarmiento brace, with no revision fixation surgery necessary to achieve union. The patient provided verbal and written consent for case report publication, including text and images. IRB was determined to be not required.

## 2. Case

**Figure 1:**
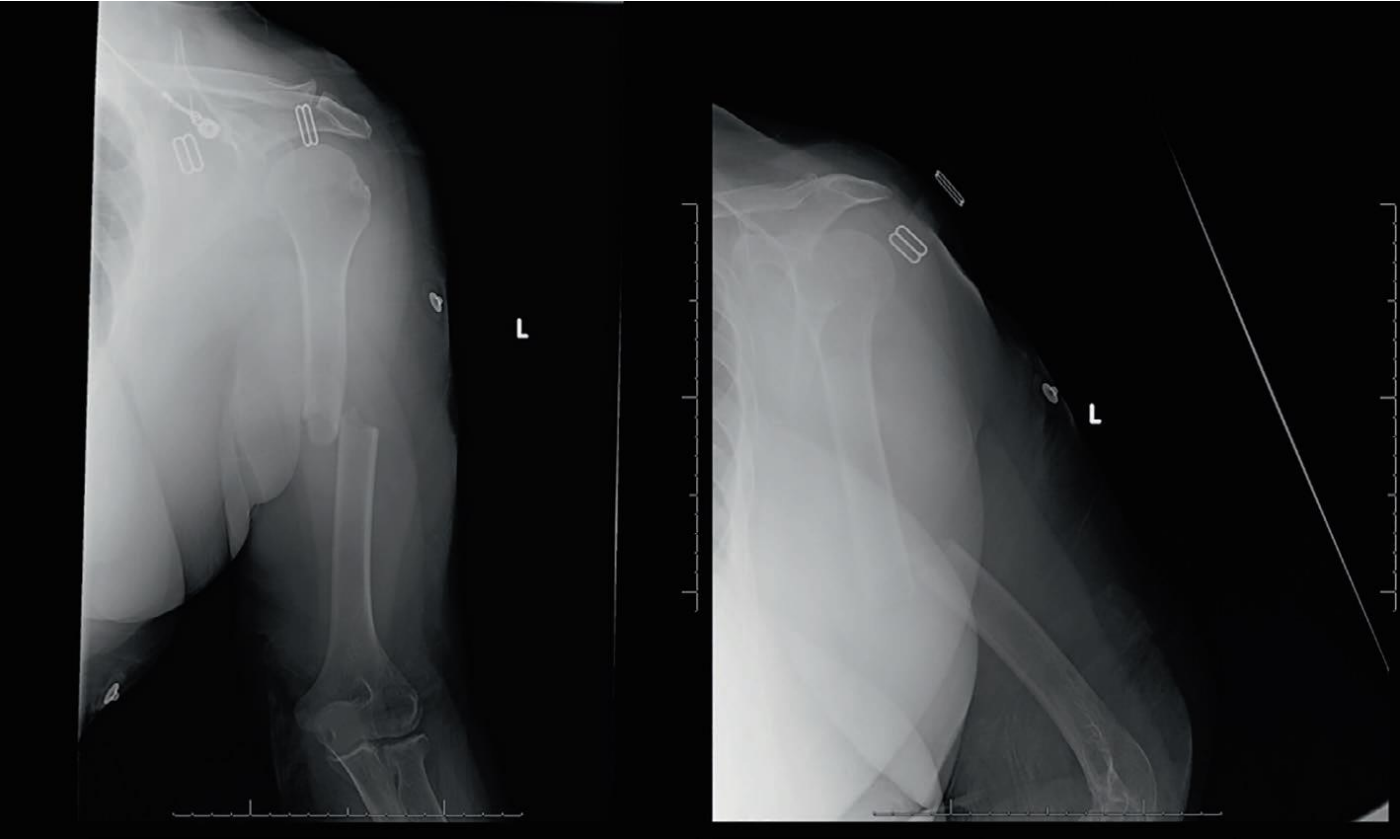
AP and lateral radiographs of the left humerus at time of initial presentation to the emergency department. Radiographs demonstrate a left midshaft humerus fracture with bayonet apposition and approximately 35° of apex anterior angulation.

The patient is a 73‐year‐old female who presented to the hospital with left arm pain and deformity after a fall. Radiographs demonstrated a left midshaft humerus fracture (Figure 1). She denied any weakness or numbness to the left upper extremity. Examination demonstrated an obvious deformity, significant swelling, and tenderness to palpation. Her past surgical history was positive for bilateral total knee arthroplasties. Her past medical history was positive for hypothyroidism and hypertension. Active medications included lisinopril, amlodipine, and Synthroid. Her BMI was found to be 28. She denied any history of tobacco or alcohol use. The patient was evaluated and placed into a size large Sarmiento brace (DJO ProCare Humeral Fracture Brace [over the shoulder], Part Number 79‐97957) in the emergency department. She was given a department handout regarding her brace (File S1), with instructions to wear the brace for 24 h per day. She was instructed to tighten the brace 2–3 times per day and informed regarding clinic follow‐up at 2 and 6 weeks in clinic. We discussed expectations including bracing for at least 6 weeks, and that an assessment would be made at 6 weeks to determine if further intervention is necessary. This assessment is based on Driesman et al.′s study regarding mobility at the fracture at 6 weeks [14]. She was counseled on the functional nature of the brace and encouraged to maintain elbow range of motion. She was also given a 1–2‐lb weight limit. She discharged with instructions to follow‐up in the outpatient orthopedic trauma clinic in 2 weeks.

**Figure 2:**
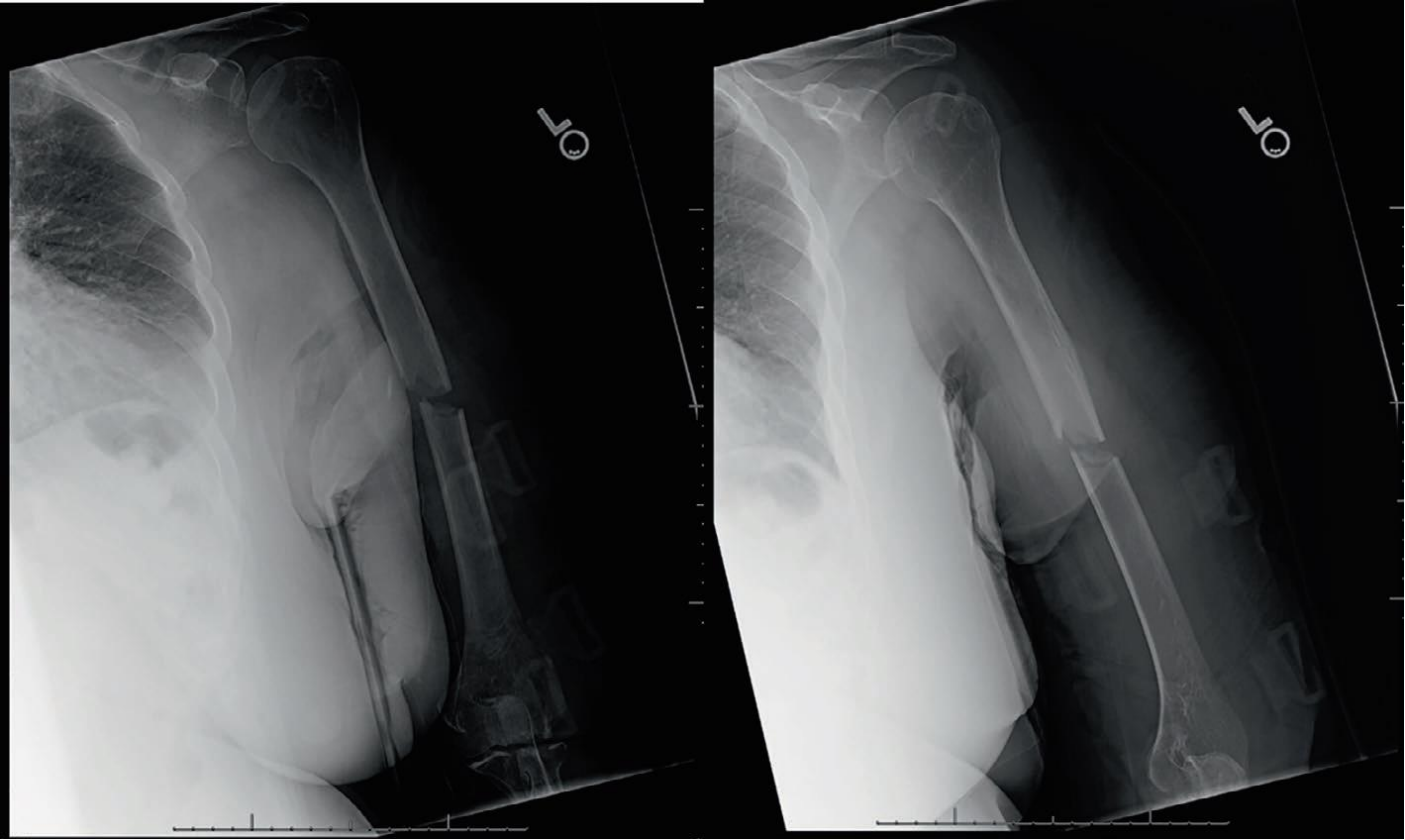
AP and lateral radiographs of the left humerus taken at the 2‐week follow‐up visit in the Sarmiento brace. Alignment is improved compared with initial injury films with some mild distraction. No sign of callous formation.

**Figure 3:**
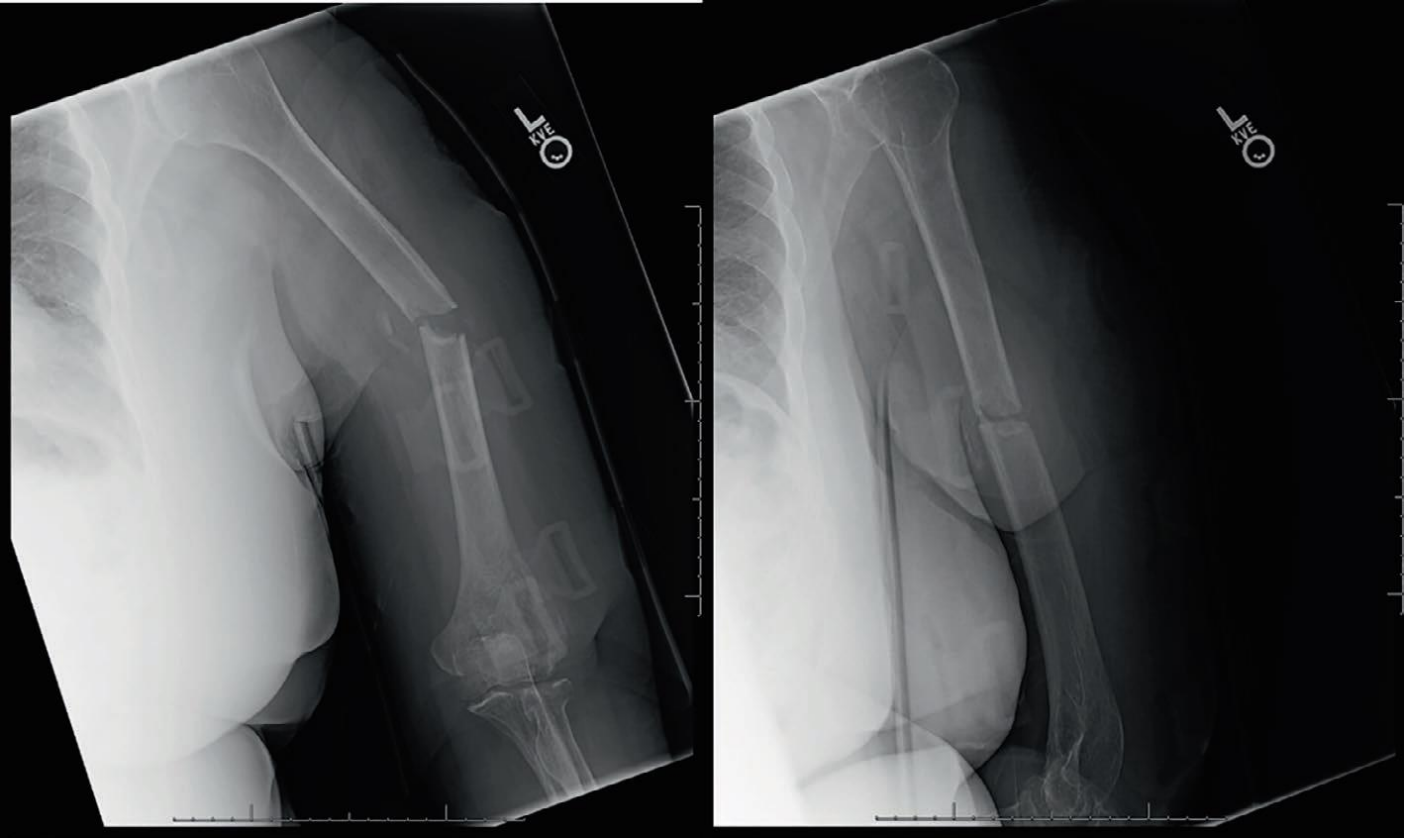
AP and lateral radiographs of the left humerus obtained 4 weeks after injury. There is approximately 35° of varus malalignment with some interval callus formation.

**Figure 4:**
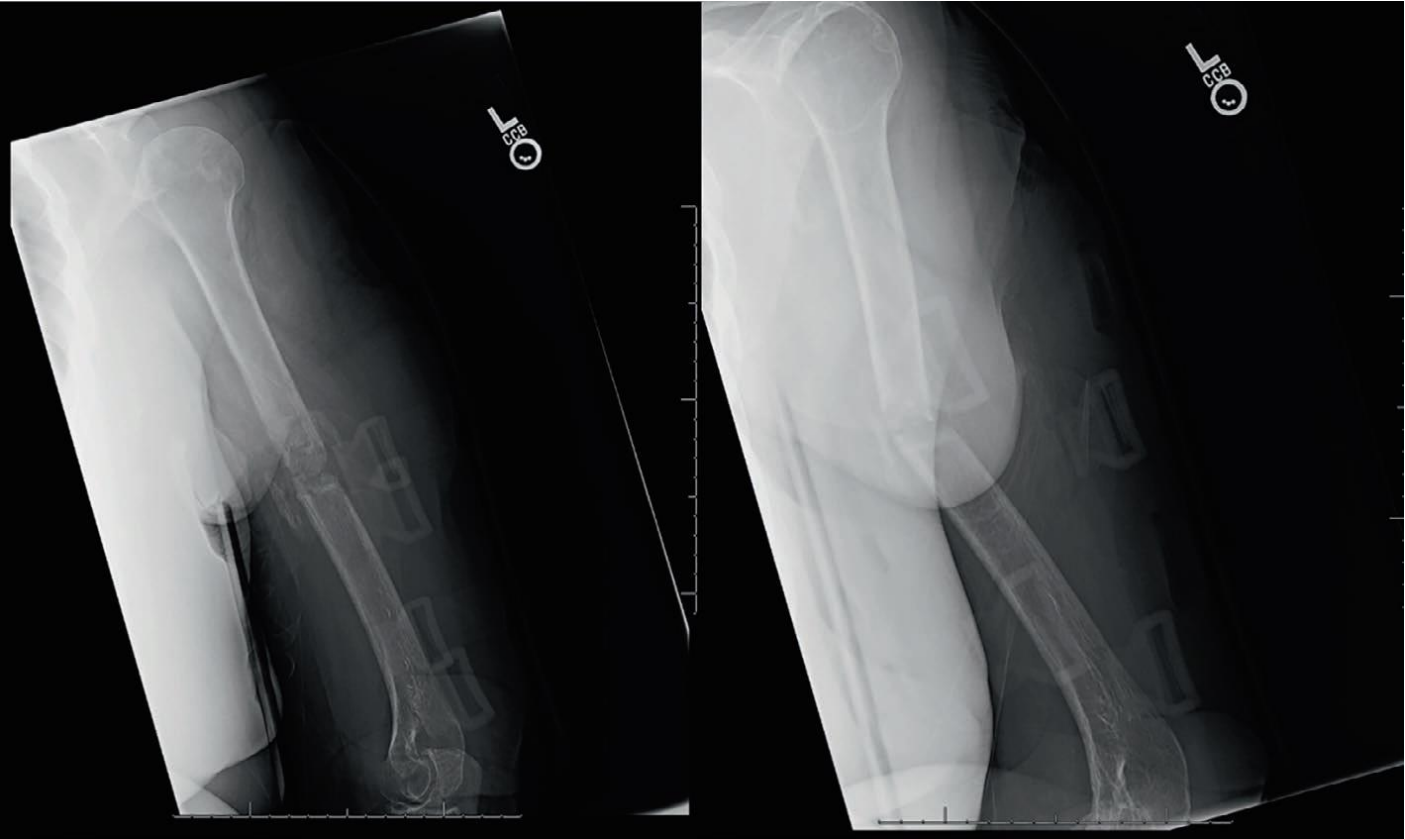
AP and lateral radiographs taken 6 weeks after injury. Radiographs demonstrate interval callus formation with a 23° apex anterior malalignment.

The patient was followed up as instructed, following our department protocol of 2‐ and 6‐week appointments. An additional appointment was made by the patient at 4 weeks due to her concern for continued pain. Figures 2, 3, and 4 demonstrate the patient′s radiographic findings at her follow‐up visits 2, 4, and 6 weeks after injury, respectively. She reported compliance with brace wearing and had been doing range of motion exercises as instructed. She had normal range of motion of her elbow and was compliant with the 1–2‐lb weight limit. Radiographs demonstrated interval callus formation with some mild angulation. The patient had notable motion at the fracture site at the 6‐week visit, and was counseled regarding the 80% chance of nonunion based on Driesman et al.′s study [14, 20]. Brace adjustments were made due to slight loosening, and the patient made the informed decision to continue nonoperative treatment in her brace.

**Figure 5:**
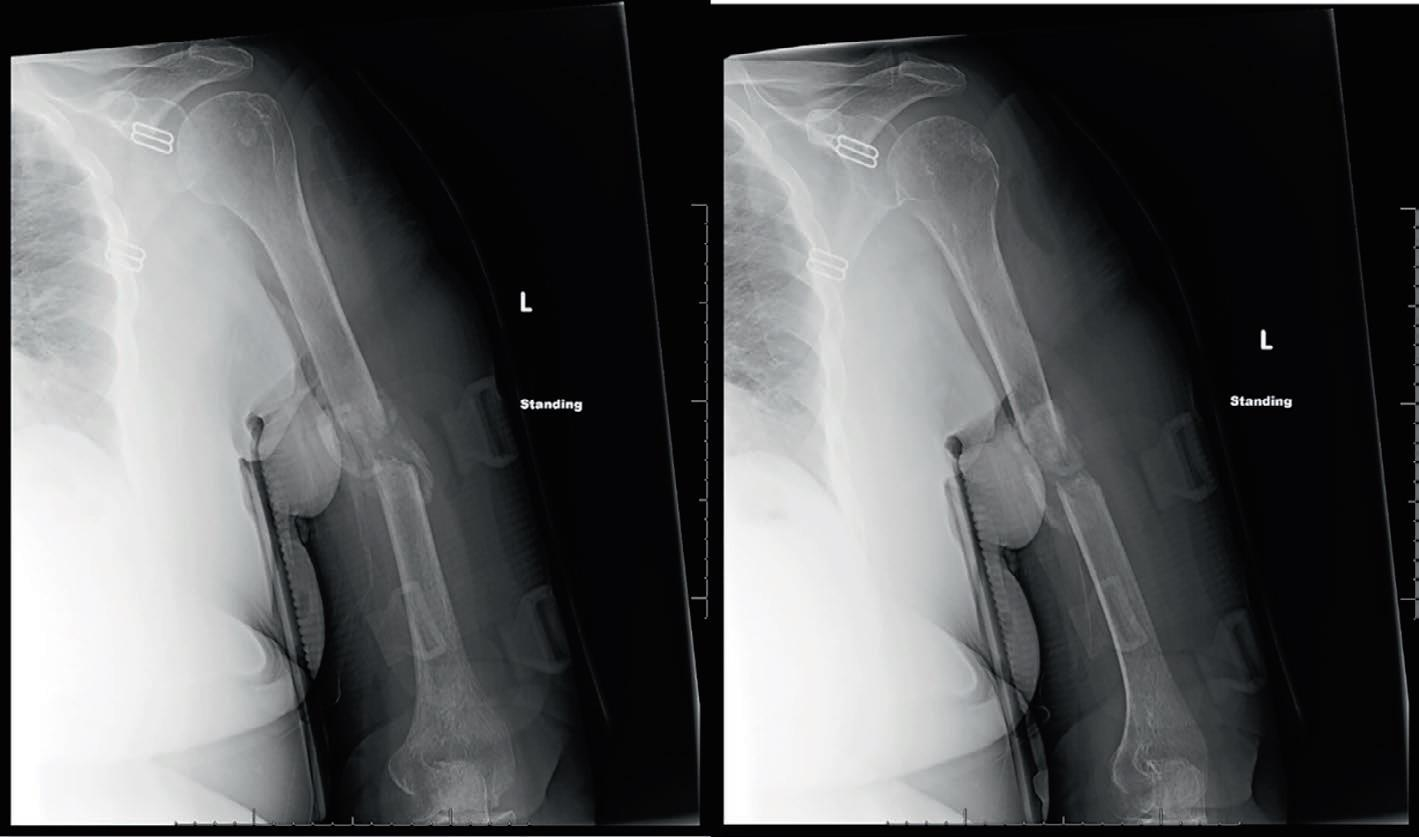
AP and lateral radiographs of the left humerus taken 12 weeks after injury demonstrating a nonunion.

The patient returned to clinic 6 weeks later, now 12 weeks out from her injury. She reported continued pain and difficulty using her arm. Examination demonstrated gross instability at the fracture site, but otherwise good range of motion of her elbow. Radiographs at this visit showed callus formation, but no significant healing (Figure 5). A diagnosis of hypertropic nonunion was made, and the patient was counseled regarding operative intervention. Nonunion labs including a complete blood count (CBC), comprehensive metabolic panel (CMP), erythrocyte sedimentation rate (ESR), C‐reactive protein (CRP), thyroid stimulating hormone (TSH), parathyroid hormone (PTH), and vitamin D levels were collected, and the patient was scheduled for surgery. The CBC, CMP, ESR, and CRP were negative for an infectious or metabolic process. Unfortunately, PTH, TSH, and vitamin D levels were never collected and are unavailable for analysis. The CMP demonstrated normal total protein, albumin, and calcium levels.

**Figure 6:**
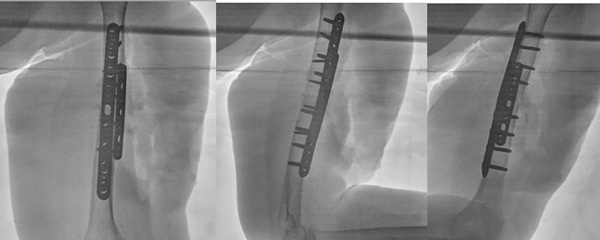
Intraoperative fluoroscopic views demonstrating reduction of the fracture with dual‐plate fixation.

The patient was taken to surgery 13 weeks after the initial injury. She was treated with a left humerus nonunion takedown and open reduction with internal fixation. A standard anterolateral approach to the humeral shaft was made and a brachialis split was utilized. Significant callus formation was noted and taken down. A 9‐hole 4.5 mm straight compression plate measuring 158.8 mm in length was placed on the anterolateral humerus. Two nonlocking screws were used to bring the plate to the bone with the distal screw placed oblique in the plate to generate compression through the plate. The remaining six screws placed were locking screws, for a total of eight screws in the plate. The working length of this plate was approximately 37 mm. An additional 7‐hole 3.4 mm straight compression plate measuring 90 mm in length was placed on the lateral humerus for added stabilization, as poor bone quality was noted. Two nonlocking screws were used to bring the plate to the bone, and two additional locking screws were placed to provide stabilization as a neutralizing plate. The two nonlocking screws were then replaced with locking screws. The working length of this plate was approximately 28 mm. Acceptable alignment was confirmed on fluoroscopic imaging, and the autograft from the callus takedown was placed as bone graft. The patient was given a 10‐lb weightbearing limit for 6 weeks and instructed to have range of motion as tolerated. Figure 6 demonstrates the position of the fracture and implants at the completion of the procedure.

**Figure 7:**
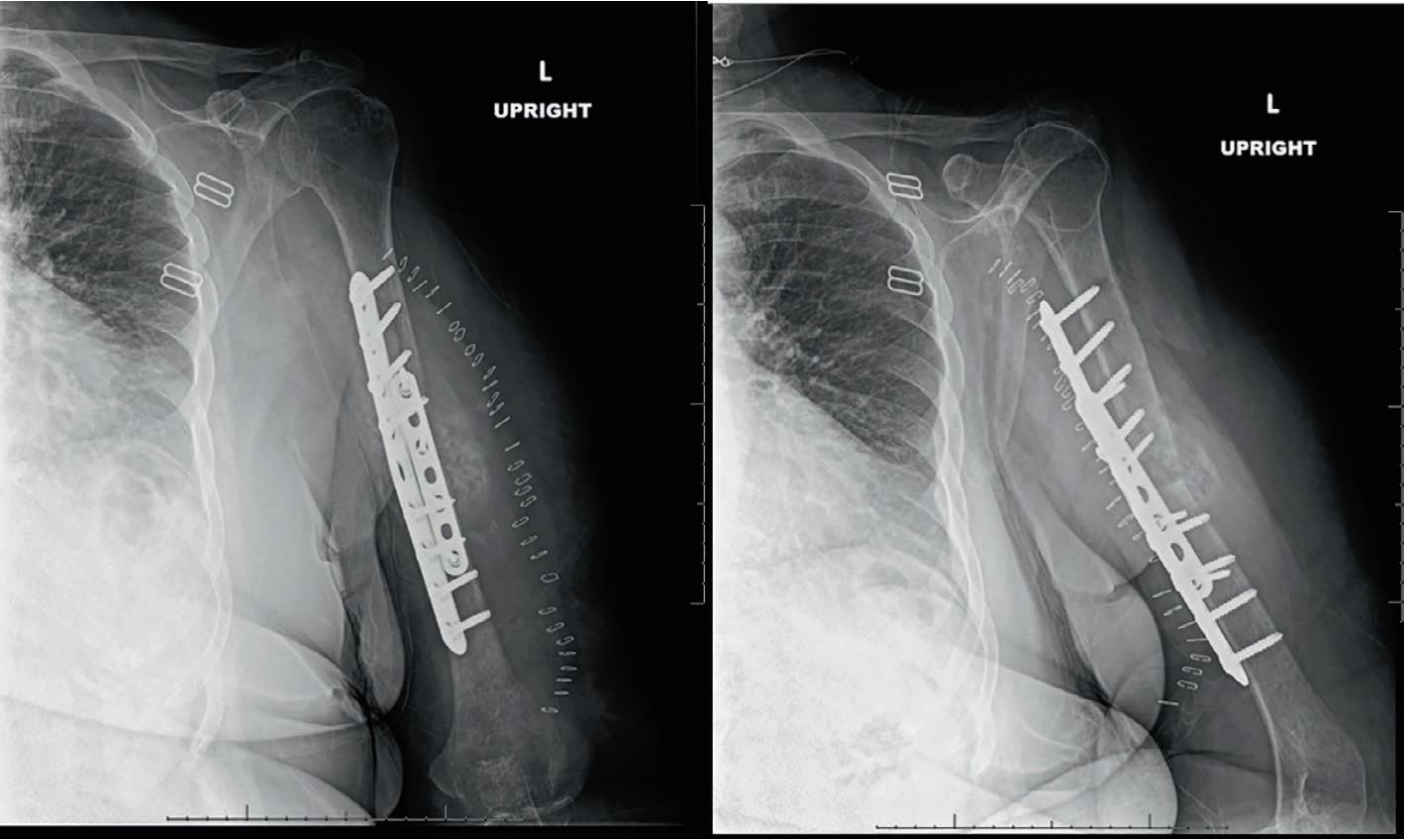
Radiographs obtained 3‐weeks postoperative demonstrating hardware failure with the large frag plate pulling out of the proximal segment and two screws pulling out of the small frag plate. There is some callus formation posteriorly. Overall, the alignment of the humerus was maintained.

The patient′s first postoperative follow‐up appointment occurred at 3 weeks postsurgery. The patient had been compliant with restrictions. Radiographs demonstrated hardware failure with plate pull‐off and loss of fixation (Figure 7). A long discussion occurred with the patient regarding revision surgery, and surgery was advised. The patient elected to return home to process this information and would call to schedule surgery.

**Figure 8:**
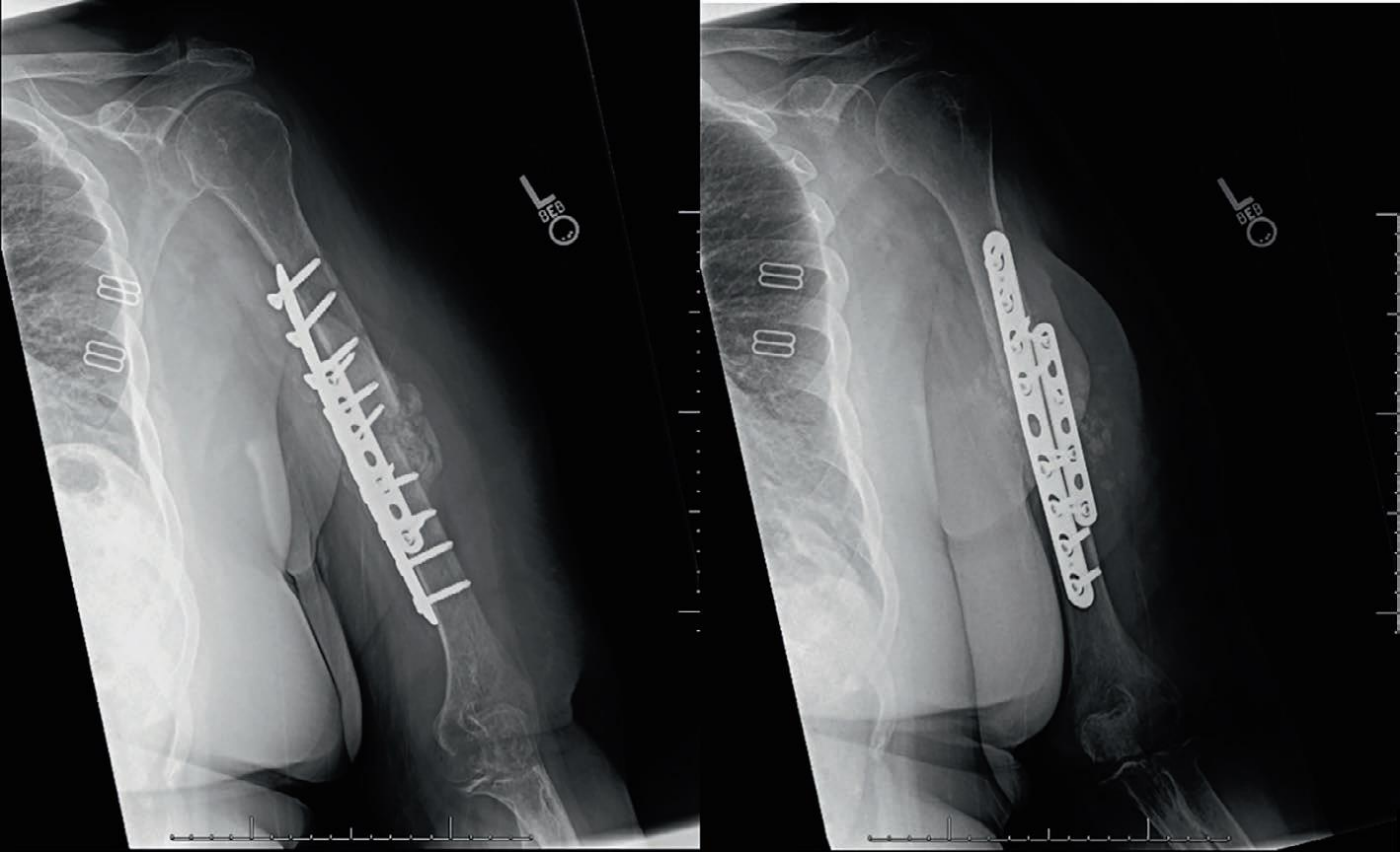
Radiographs at 5 weeks postoperatively demonstrate improved alignment and callus formation. The plate and screws are not pulling off the plate any further.

The patient returned to clinic 2 weeks later, now 5 weeks post‐op from surgery. The patient had started wearing her Sarmiento brace again and had been actively working on elbow flexion and extension. The decision to return to the brace was made by the patient and was not made with provider assistance or counseling. She had no clinical deformity. Radiographs demonstrated improved alignment from her previous visit (Figure 8). The plate had not pulled off the bone any further, and she had increased callus formation. She had elbow range of motion from 0° to 140° of flexion. Active forward elevation at the shoulder to 80°, active abduction to 40° and passive abduction to 90° with no gross mobility at the fracture site. The decision was made to continue treatment in the Sarmiento brace.

**Figure 9:**
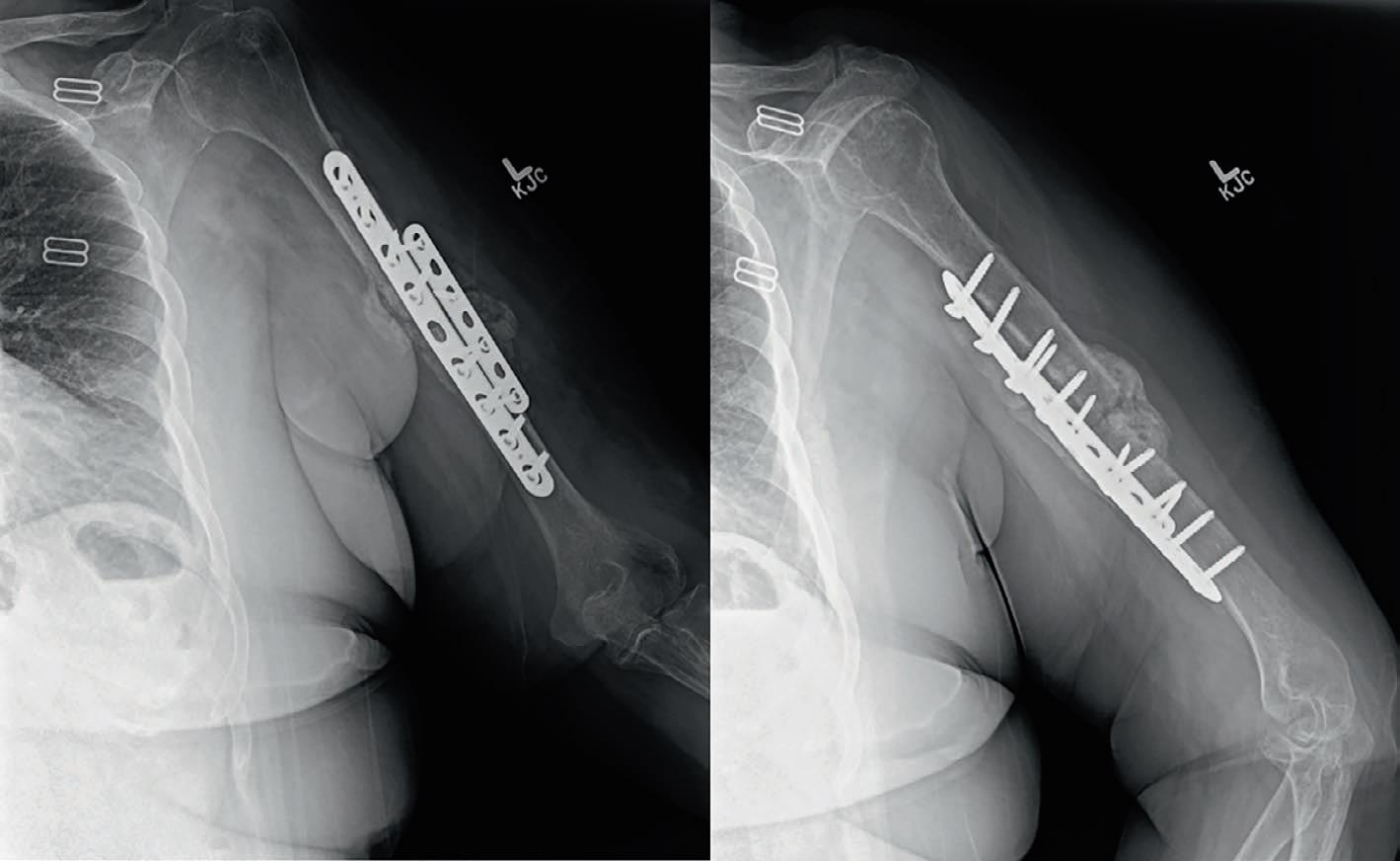
Radiographs taken 2 months postoperatively demonstrate acceptable alignment and improved callus formation from prior radiographs. Union of the fracture appears to be achieved.

Two months after surgery, the patient demonstrated improved function and decreased pain. She had continued to wear her Sarmiento brace for comfort. Her active shoulder forward flexion had increased to 110° and her active abduction increased to 90°. Radiographs demonstrated acceptable alignment with increased callus formation. Her fracture appeared to have achieved union at this time (Figure 9). She was advised to discontinue the use of the Sarmiento brace. She was referred for physical therapy and instructed to follow‐up 3 months later.

**Figure 10:**
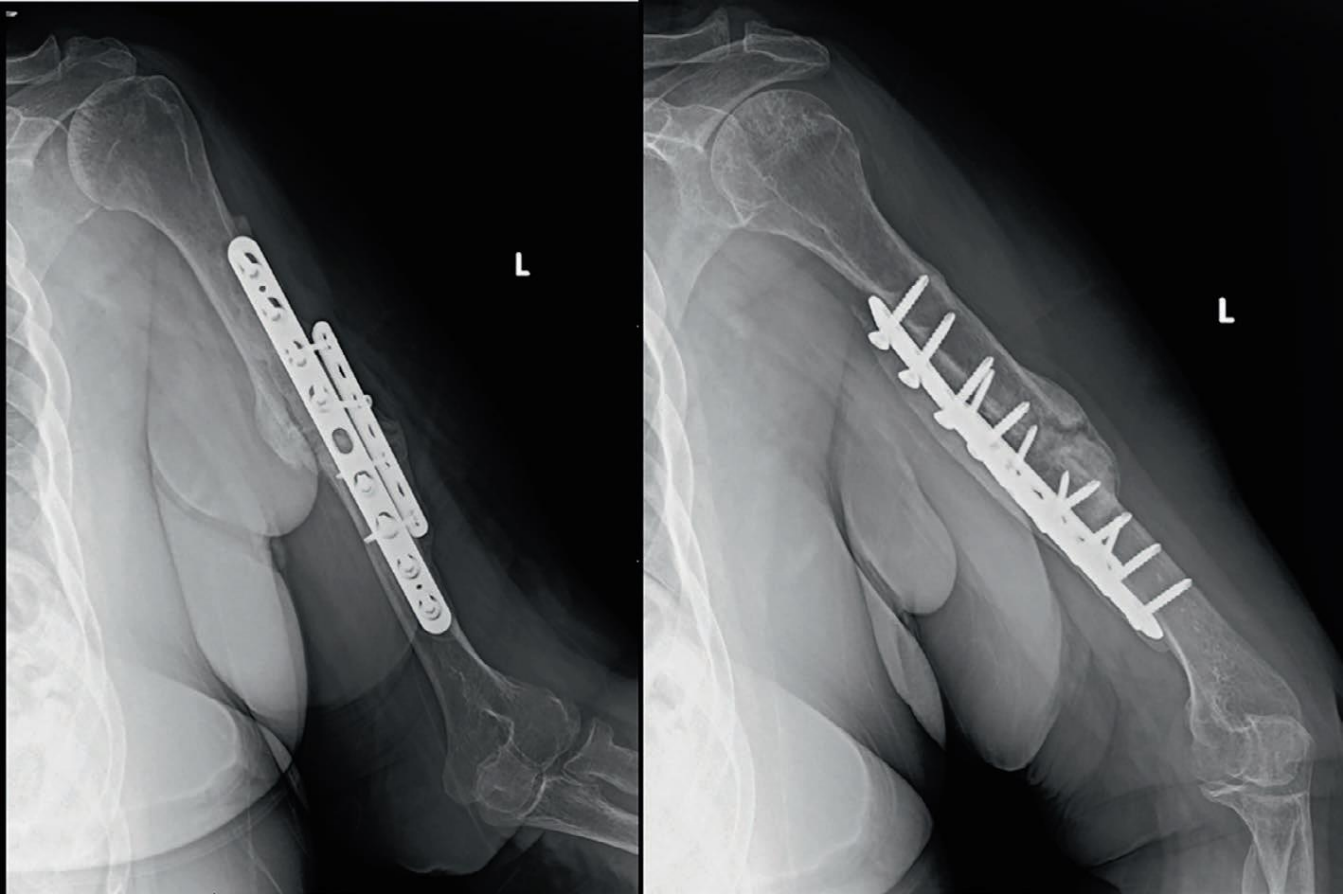
Radiographs taken 5 months postoperatively, 8 months after the initial injury demonstrating a healed humerus fracture with acceptable alignment.

The patient presented for her 5‐month postoperative clinic visit, which was now 8 months after her initial injury date. She reported visual analog scale (VAS) pain as a 2 out of 10. Elbow and shoulder range of motion was symmetric to the contralateral side and included 160° of forward flexion, abduction to 150°, 40° of external rotation at the side, and internal rotation to her lumbar spine. Radiographs demonstrated a healed humeral shaft fracture with abundant callus formation (Figure 10). Other functional scores were not assessed. She was happy with her arm and was given a full release to activities as tolerated. She was instructed to follow up as needed.

## 3. Discussion and Conclusion

Brinker et al. demonstrated that metabolic and endocrine abnormalities can contribute to nonunion, and some patients can heal their nonunion with medical treatment alone [20]. Although we suspect that fracture instability was the cause of fracture nonunion in this patient, the role of metabolic and endocrine abnormalities cannot be forgotten. This patient has a history of hypothyroidism that has been well maintained on Synthroid. Nonunion labs were ordered as recommended by Brinker et al. but were not obtained [20].

The lack of TSH, PTH, and vitamin D nonunion labs represents a major limitation of this paper. We do not know how the patient′s metabolic status influenced the initial nonunion development, or the subsequent healing of her fracture after resuming brace wear. This is compounded by the patient′s known history of a thyroid disorder. It is well known that metabolic status plays a part in healing. Brinker et al. demonstrated that metabolic and nutritional status are central contributors to bone healing and nonunion development [20]. The patient demonstrated some callus formation and was diagnosed with a hypertrophic nonunion. Although hypertrophic nonunion is primarily attributed to insufficient stability, some studies have demonstrated that metabolic dysfunction can coexist and further compromise bone healing [21–24]. Bergin et al. demonstrated that up to 75% of fracture nonunions may have some form of metabolic dysfunction regardless of nonunion type [24]. Identifying and correctly addressing any metabolic dysfunction may have avoided an unnecessary surgery for this patient. It is also possible that some metabolic correction occurred after the surgical failure and contributed to her fracture healing in the brace. The nonunion labs were ordered, but the patient did not have these drawn. The lab is in the same building as our clinic, and our typical protocol is to have the labs drawn as the patient is leaving their appointment. Our department is considering options to improve this and develop a better system to ensure that necessary labs are completed if a patient is unable to get them drawn after their appointment.

The primary goal of surgical stabilization of humeral shaft fractures is to achieve a stable fixation that allows for early range of motion exercises [25, 26]. Common surgical techniques to treat humeral shaft fracture nonunions include open reduction internal fixation with plate fixation and intramedullary nailing [19]. Both techniques typically provide adequate stabilization for bone healing. Sufficient stabilization obviates the need for additional external bracing such as a Sarmiento brace, and thus bracing after surgical stabilization has not been supported in the literature.

In this case, our patient successfully avoided a second surgery by returning to Sarmiento brace wear. We hypothesize that the patient′s body habitus, fracture pattern, and poor bone quality contributed to her nonunion [27]. Given the hypertrophic nature of her nonunion, we felt that the additional stability imparted by internal fixation would lower the strain at the fracture site to allow for union. Unfortunately, inadequate stability was achieved during surgery, leading to a failure of her initial surgery. We postulate that poor bone quality and continued motion at the fracture site contributed to her fixation failure after surgery. We tried to anticipate this by adding the additional 3.4‐mm plate on the lateral humerus. There are some additional technical considerations that could have contributed to the inadequate stability. This could include poor surgical technique, inadequate screw purchase, or lack of appropriate locking screw supplementation. The leading author maintains that a good surgical reduction was obtained and that stability was thought to be achieved at the time of surgery after the placement of both plates and primarily using locking screws. Nevertheless, adequate stability was not achieved until she went back into the brace on her own accord. What fixation was achieved intraoperatively may have been enough to supplement the stabilization of the brace. Another possibility is that the lead up to surgery, and the recovery period, provided some enhancement to the patient′s metabolic status. A more complete understanding of the nonunion process, and how metabolism and stability interplay with each other, may be needed to fully understand the delay and cause of this patient′s union.

The concept of utilizing a Sarmiento brace after surgical stabilization for humeral shaft fractures is interesting and has not been previously described in the literature. Functional bracing following failed surgical stabilization may be an option for the right patient under the right circumstances. Our experience indicates that brace utilization after surgical stabilization failure would best be used in the case of poor bone quality and questionable intraoperative stability in the setting of a noninfected hypertrophic nonunion with acceptable alignment. Revision surgery remains the standard, and further research is needed to assess functional bracing as an alternative to revision surgery. Further research regarding the stability that can be achieved via a Sarmiento brace is needed as well, as a combination of surgical fixation and functional brace supplementation may be a useful strategy for certain patients.

## Funding

No funding was received for this manuscript.

## Conflicts of Interest

The authors declare no conflicts of interest.

## Supporting information


**Supporting Information** Additional supporting information can be found online in the Supporting Information section. File S1:Sarmiento brace handout provided to all humeral shaft fracture patients treated with a Sarmiento brace.

## Data Availability

The data that support the findings of this study are available on request from the corresponding author. The data are not publicly available due to privacy or ethical restrictions.
