# Supplementary material for: Utilizing a Sarmiento Brace to Attain Union of a Humeral Shaft Fracture Nonunion With Hardware Failure
Source: Case Rep Orthop. 2026 Apr 25;2026:9111506. doi: 10.1155/cro/9111506 (PMC13110351; doi:10.1155/cro/9111506)
Supplement: Supplementary file 1 — Supporting Information Additional supporting information can be found online in the Supporting Information section. File S1:Sarmiento brace handout provided to all humeral shaft fracture patients treated with a Sarmiento brace. [file CRO-2026-9111506-s001.pdf]

FUNCTIONAL BRACING (SARMIENTO) FOR HUMERUS FRACTURES

WHAT IS A HUMERAL FRACTURE ORTHOSIS (SARMIENTO BRACE)?

A humeral fracture orthosis (Sarmiento brace) is designed to immobilize a humerus (upper arm bone) fracture to assist with healing. The brace should be worn 24 hours a day for 6 weeks or longer.

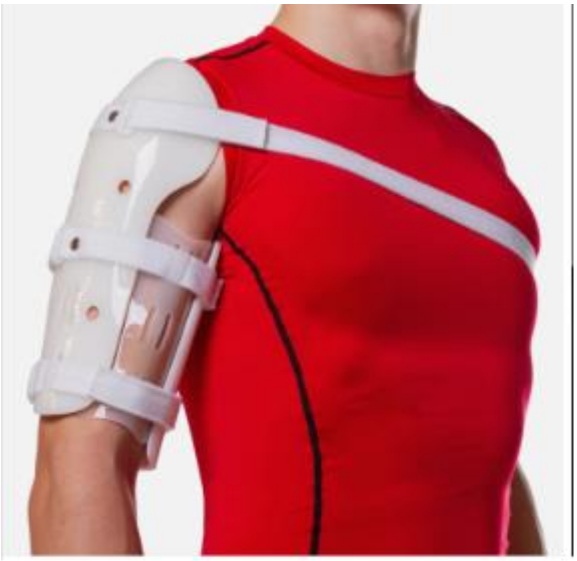

HOW SHOULD THE BRACE FIT?

The brace works by applying circumferential pressure around the bone to prevent it from moving. In order to work, the brace must be tight so it is important to adjust the brace 2-3 times a day, especially early on as swelling improves. Additionally, for the brace to work, you must use your elbow which activates the muscles and brings the bone back to the proper position.

WASHING AND HYGIENE

Your brace needs to remain dry at all times which means sponge baths are typically recommended while using the brace. If your arm or the brace gets wet, it should be removed and dried while keeping the arm in a comfortable position.

FOLLOW UP WITH YOUR BRACE

Once your brace is applied, you will be seen at 2 weeks and 6 weeks in clinic. If your fracture is not showing signs of healing at 6 weeks, surgery may be indicated. However, studies show up to 80% of patients can be treated successfully without surgery.

WHEN TO CALL YOUR DOCTOR

If you are having skin irritation, loss of sensation, or worsening swelling after the first week, you should call your doctor to be assessed. Our clinic can be reached at (405)-271-2663 or (405)-271-4906
